# *In vitro* anti-inflammatory activity of *Plantago major* L. and *Piper aduncum* L. on phospholipase A2 from the venom of snake *Lachesis muta muta*

**DOI:** 10.17843/rpmesp.2023.403.12191

**Published:** 2023-09-26

**Authors:** Mirtha Yarleque-Chocas, Flor Dorregaray-Llerena, Armando Yarleque-Chocas, Celso Gonzales-Chavesta

**Affiliations:** 1 Biochemistry and Natural Active Principles Research Laboratory, Hipólito Unanue Faculty of Medicine, Universidad Nacional Federico Villarreal, Lima, Peru. Universidad Nacional Federico Villarreal Biochemistry and Natural Active Principles Research Laboratory Hipólito Unanue Faculty of Medicine Universidad Nacional Federico Villarreal Lima Peru; 2 Plant Genetic Resources Laboratory, Faculty of Agricultural Sciences, Universidad Agraria del Ecuador., Milagro, Ecuador. Universidad Agraria del Ecuador Plant Genetic Resources Laboratory Faculty of Agricultural Sciences Universidad Agraria del Ecuador Milagro Ecuador; 3 Laboratory of Molecular Biology, Faculty of Biological Sciences, Universidad Nacional Mayor de San Marcos, San Marcos National University, Lima, Peru. Universidad Nacional Mayor de San Marcos Laboratory of Molecular Biology Faculty of Biological Sciences Universidad Nacional Mayor de San Marcos Lima Peru; 4 Department of Statistics and Informatics, Universidad Nacional Agraria la Molina, La Molina, Lima, Peru. Universidad Nacional Agraria La Molina Department of Statistics and Informatics Universidad Nacional Agraria la Molina Lima Peru

**Keywords:** Plant extracts, Plantago, Piper, Venom, Phospholipase A2, Anti-inflammatory

## Abstract

**Objective.:**

To evaluate the in vitro inhibitory activity of Plantago major “llantén” and *Piper aduncum* “matico” extracts on phospholipase A2 (PLA2) from the venom of the snake *Lachesis muta muta*.

**Materials and methods.:**

We carried out an explanatory study with experimental design. Leaves of *P. major* and P*. aduncum* were collected in the province of Huarochirí in Lima, Peru. Then, we prepared alcoholic extracts diluted in distilled water and conducted phytochemical assays, quantification of phenols and flavonoids, thin layer chromatography (TLC) on cellulose and enzymatic activity with PLA2. The ability to inhibit PLA2 with the extracts under study and their fractions was analyzed. The Kruskal Wallis test and Bonferroni multiple comparisons were used during statistical analysis.

**Results.:**

Phenols, flavonoids and tannins were qualitatively identified in both *P. major* and *P. aduncum;* in addition, *P. aduncum* presented saponins. The inhibition of PLA2 activity of the venom by the total extract of P. major was 45.3%, and its fractions showed the following inhibition values: 31.1% for LLF-1, 66.3% for LLF-2 and 65.5% for LLF-3. The inhibition values for the total extract of *P. aduncum* were 86.9%, and its fractions showed the following inhibition rates: 34.3% for MF-1, 67.1% for MF-2 and 54.9% for MF-3. Statistical analysis showed significant differences in the inhibition of PLA2 (p=0.009) by the extracts.

**Conclusion.:**

The tests demonstrated an association between the anti-inflammatory effect of the extracts and PLA2 inhibition.

## INTRODUCTION

Peru is a country with a great wealth of phytogenetic resources, with a total of 19,147 species of vascular plants alone. The plant diversity contributes to the traditional knowledge of the country’s human communities, which actively participate in their conservation and sustainable use ^1^. Plant species sustain diverse ecosystems, and many of them have not yet been fully studied, although their empirical use is widespread, particularly for their medicinal properties.

In this context, *Piper aduncum* L., commonly known as “matico”, is a native species of Peru and is distributed in several departments, including Amazonas, Ayacucho, Cajamarca, Cuzco, Huánuco, Junín, Lambayeque, Lima, Loreto, among others ^2^. On the other hand, *Plantago major* L., a species introduced from Eurasia, is found in tropical and subtropical areas throughout the world, including Peru, where it is distributed in all geographic regions and at altitudes between 1500 and 2000 m ^3^.

It is interesting to identify the ethnomedicinal uses of *P. aduncum* and *P. major* in Peru, where the fresh or dried leaves are used as an infusion to treat a variety of health problems, such as colds, fungal infections, coughs, wounds, bronchitis, fever, kidney, lung, gastric and respiratory problems, as well as to wash inflamed wounds ^4^.

Certain flavonoids, found I some plants, may have anti-inflammatory properties, which is why it is important to study them. Blocking inflammation implies the ability to act on the activity of enzymes involved in the synthesis of thromboxanes and leukotrienes, such as phospholipase A2 (PLA2) and cyclooxygenases (COXI and COXII), as well as having an inhibitory effect on the production of nitric oxide (NO), a cellular mediator involved in physiological and pathological processes related to inflammation ^5^.

The venom of the snake *Lachesis muta muta*, which inhabits the Peruvian jungle and is known as “shushupe”, has high PLA2 activity. For this reason, the search for inhibitors of this enzyme is proposed as an alternative to understand the mechanism of the anti-inflammatory process ^6^.

This study aimed to evaluate the *in vitro* inhibitory activity of *P. major* “llantén” and *P. aduncum* “matico” extracts on PLA2 from the venom of the snake *Lachesis muta muta*, since this enzyme plays an important role in the inflammatory process. It is important to emphasize that this study represents the first contribution at national level in the knowledge of the action of the compounds contained in these plants in the inhibition of PLA2, that is, in their anti-inflammatory capacity.

**KEY MESSAGES**

Motivation for the study. *P. major* “llantén” and *P. aduncum* “matico” have been used in traditional medicine for their anti-inflammatory effects, and we aimed to investigate these effects in relation to the PLA2 enzyme that initiates the inflammatory process.

Main findings. The extracts from the two plants inhibit PLA2 of snake venom; the extract from *P. aduncum* showed to be more efficient. The fractions showed different degrees of inhibition.

Implications. The extracts of *P. major* and *P. aduncum* and their fractions showed in vitro inhibitory effects on snake venom PLA2, which is related to the anti-inflammatory effect.

## MATERIALS AND METHODS

This was an explanatory and experimental research. It was conducted at the Biochemistry and Natural Active Principles Research Laboratory of the Faculty of Medicine “Hipólito Unanue” of the Universidad Nacional Federico Villarreal from July 2021 to July 2022.

### Plant samples

The leaves of *Piper aduncum* L. and *Plantago major* L. were collected between August and October 2019 in the province of Huarochirí in Lima, Peru. This locality is located at an altitude of 3044 MASL (11°50′41′S 76°23′02′W), with a temperature ranging from 15 °C to 21 °C.

The species were taxonomically identified in the Herbarium of the Faculty of Natural Sciences of the Universidad Nacional Federico Villarreal, where the samples were deposited with accession codes No. 7400 for *Piper aduncum* L. and No. 7399 for *Plantago major* L.

### Snake venom

The freeze-dried venom of the Peruvian snake *L. muta muta* was provided by the Laboratory of Molecular Biology, which is in charge of the “Oswaldo Meneses Serpentarium” of the Museum of Natural History of the Universidad Nacional Mayor de San Marcos in Lima, Peru. The specimens of *L. muta muta* found in the serpentarium were collected from the Alto Marañón area, in the department of Amazonas, and are kept in captivity, complying with the norms established by the National Forestry and Fauna Service (SERFOR) of the Ministry of Agriculture and Irrigation.

### Alcoholic extracts

The samples consisted of dried leaves of each species. Two hundred grams of each sample were collected and pulverized to increase their surface area in contact with the solvent. Then, these pulverized samples were placed in amber-colored jars for maceration with 96° ethanol for seven days. After the maceration period, the extracts were filtered to remove solid particles and obtain a clarified liquid. Subsequently, the ethanol solvent was evaporated in a rotary evaporator until the extracts were completely dry.

### Qualitative phytochemical assays

The following qualitative phytochemical assays were carried out to determine the presence of different compounds in the extracts of *Plantago major* and *Piper aduncum*:

Determination of total phenols: the ferric chloride reagent was used to form a dark blue colored complex in the presence of phenolic compounds. A 1 mL solution of the sample in 70% ethanol, and 2 drops of 1% ferric chloride were added in a test tube, and the color changed ^7^.

Evaluation of flavonoids: the oxidation reaction of metallic magnesium with concentrated hydrochloric acid was used to form magnesium chloride, which reacts with the flavonoids and generates colored complexes. We placed 1 mL of ethanolic solution of the sample in a test tube, then 10 drops of concentrated HCl and magnesium metal chips were added. After resting for 5 minutes, we observed the color of the reaction ^7^.

Identification of saponins: saponins have the ability to break the surface tension of water and form foam. We added 1 g of dry extract to 10 mL of distilled water, then it was heated at 100 °C for 5 min and shaken vigorously. Persistent foaming after 5 min was scored with crosses, indicating the presence of saponins ^7^.

Tannin recognition: tannins have the ability to precipitate proteins. We placed 1 mL of 1% gelatin in saline solution in a test tube, to which 200 uL of the sample dissolved in distilled water were added. The presence of protein precipitation was scored as positive, which indicated the presence of tannins ^7^.

### Quantification of phenols

The Folin-Ciocalteu reagent ^8^ was used for the colorimetric oxidation-reduction reaction. We added 1 mL of 10% Folin-Ciocalteu to 0.1 mL of each sample, then it was left to rest for five minutes, then 1 mL of 7.5% sodium carbonate was added. The tubes were then placed in a water bath (45°C) for 15 minutes and read at 765 nm in a Thermo Scientific Genesys 10 spectrophotometer. The results were compared with a gallic acid curve (Sigma Chemical) and expressed in mg/g of sample. Three replicates were performed with each sample.

### Quantification of flavonoids

Total flavonoids were analyzed by a spectrophotometric method ^9^. We mixed 250 µL of each sample with 1000 µL of deionized water, then 75 µL of 20% NaNO_2_ was added, allowed to react for five minutes and then 75 µL of 10% AlCl_3_ and 500 µL of 1 M NaOH were added. The mixture was centrifuged at 3500 rpm for five minutes. Total flavonoids were expressed as catechin mg/g sample. Absorbance was measured at 510 nm. Three replicates were performed with each of the plant samples.

### PLA2 activity

We determined PLA2 activity by measuring the clotting time of a 45% egg yolk emulsion with 10 mM Tris HCl buffer and 10 mM calcium chloride pH 7.4, after incubation with 0.125 mg/mL of *L. muta muta* venom for 10 min at 37 °C ^6^.

### Inhibition of PLA2 by the extracts

Sample preparation: 1 mL of the venom of concentration 0.250 mg/mL was collected and mixed with 1 mL of each of the plant extracts separately. These mixtures were pre-incubated for 10 min at a temperature of 37 °C.

Substrate preparation: A 45% egg emulsion was prepared in 10 M Tris HCl buffer pH 7.4 with 10 mM calcium chloride; 1.5 mL of the emulsion was used as substrate.

Incubation with substrate: 200 uL of the venom plus extract mixture was added to the substrate. Then, this mixture was heated at a temperature of 100 °C.

Measurement of coagulation time: The coagulation time of the egg yolk was measured in minutes after incubation with the venom plus extract mixture.

Calculation of specific activity: The specific activity of PLA2 was expressed as the variation of yolk clotting time per minute divided by the protein content of the venom in milligrams (U/mg).

### Thin layer chromatography on cellulose

Aqueous solutions of 0.5 g/mL of the extracts of *P. major* L. and *P. aduncum* L. were seeded on cellulose chromatographic plates, ethanol and water in concentrations of 1:2 and 1:3, respectively, were used as mobile phase.

### Statistical analysis

R v.4.2.1 software was used to analyze the existence of significant differences in the inhibitory activity on PLA2 between the total extracts and their fractions for the two species, *P. major* (LLF-1, LLF-2 and LLF-3) and *P. aduncum* (MF-1, MF-2 and MF-3) respectively, using the Kruskal-Wallis test, which is equivalent to a complete randomized design. Similarly, in order to determine which sample exhibited higher inhibitory activity on PLA2, pairwise comparisons were carried out using the PLA2 activity values of the venom and each of the plant samples; the Bonferroni multiple comparisons test was also applied. This choice was based on the fact that the assumptions of variance analysis (normality of errors and homogeneity of variances) were not met in both cases.

To validate the results, we used SPSS version 28 software d and multiple comparison plots were generated to provide a more detailed explanation of the comparisons between pairs of samples.

### Ethical Aspects

The project was evaluated by the Ethics Committee of the Research, Innovation and Entrepreneurship Unit of the Faculty of Natural Sciences and Mathematics of the Universidad Nacional Federico Villarreal (code No. 002-2022).

## RESULTS

### Detection of bioactive components in total extracts

Table 1 shows the content of phenols and flavonoids in the total extracts of *P. major* and *P. aduncum*. The total content of phenols and flavonoids present in *P. major* was lower by approximately 50% with respect to *P. aduncum*.

**Table 1:** Concentration of total phenols and flavonoids in total extracts.

Total extracts	Total phenols mg/g sample (SD)	Flavonoids mg/g sample (SD)
*P. major*	11.65 (0.037)	0.30 (0.018)
*P. aduncum*	21.12 (0.003)	0.73 (0.027)

SD: standard deviation.

As for the qualitative analysis, the extracts of *P. major* and *P. aduncum* showed a positive reaction for phenols, flavonoids and tannins; additionally, saponins were found in *P. aduncum* (Table 2).

**Table 2:** Phytochemical tests of extracts and fractions of *P. major* and *P. aduncum*.

Sample	Phenols	Flavonoids	Saponins	Tannins	PC
*P. major*	+	+	-	+	NA
LLF-1	+	+	-	+	0.58
LLF-2	+	-	-	+	0.70
LLF-3	+	+	-	+	0.88
*P. aduncum*	+	+	+	+	NA
MF-1	+	+	-	+	0.23
MF-2	+	+	-	-	0.58
MF-3	+	+	+	-	0.74

Presence (+), absence (-), PC: partition coefficient, NA: not applicable.

LLF-1: fraction 1 of *P. major*, LLF-2: fraction of *P. major*, LLF-3: fraction 3 of *P. major*.

MF-1: fraction 1 of *P. aduncum.* LLF-2: fraction 2 of *P. aduncum*. LLF-3: fraction 3 of *P. aduncum*.

### Analysis of the fractions of the extracts of *P. major* and *P. aduncum*

There were three fractions, from the TLC, of both total extracts of *P. aduncum* and *P. major*. The partition coefficient (PC) values obtained for the fractions of *P. aduncum* were 0.23 to 0.74, and 0.58 to 0.88 for the fractions of *P. major*. The phytochemical analysis of the *P. major* fractions, showed phenols, flavonoids and tannins in all of them, except for LLF-2, which did not show flavonoids. Phenols and flavonoids were found in the fractions of *P. aduncum*, additionally tannins were found in MF-1 and saponins in MF-3 (Table 2).

### PLA2 inhibitory activity of PLA2 by extracts of *P. major* and *P. aduncum*

The results of *in vitro* assays of the inhibitory effect of phospholipase A2 of *L. muta muta* venom by *P. major* and *P. aduncum* extracts showed significant variations (p=0.009).

**Figure 1:**
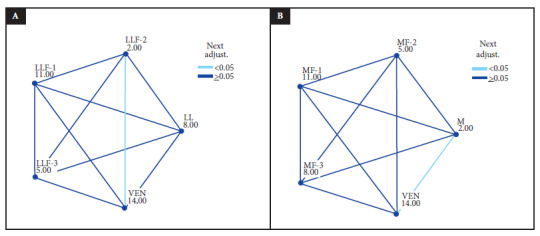
Multiple comparisons of the inhibitory enzymatic activity on PLA2 of *L. muta muta* venom.

Table 3 shows the inhibitory effect of PLA2 of *L. muta muta* venom by *P. major* total extract at a concentration of 0.5 g/mL and indicates that there are highly significant differences (p=0.009). On the other hand, Figure 1A shows the pairwise comparison of the values of PLA2 enzyme activity of the venom and those of the total extract of *P. major* and each of its fractions. We found that LLF-2 significantly (p<0.05) inhibited the PLA2 activity of the venom compared to the total extract and the other fractions.

**Table 3:** Inhibitory effect of *P. major* extract and its fractions.

Treatment	Specific activity (SD)	p-value ^a^	Inhibition %
*L. muta muta* 0.125 mg/mL	3.69 (0.002)	0.009	NA
*P. major* 0.5 g/mL	2.02 (0.002)		45.3
LLF-1 0.5 g/mL	2.55 (0.013)		31.1
LLF-2 0.5 g/mL	1.24 (0.002)		66.3
LLF-3 0.5 g/mL	1.28 (0.004)		65.5

LLF-1: fraction 1 of *P. major*, LLF-2: fraction 2 of *P. major*, LLF-3: fraction 3 of *P. major*, SD: standard deviation, NA: not applicable.

a Kruskal Wallis test.

Table 4 shows the inhibitory effect of PLA2 of *L. muta muta* venom by the total extract of *P. aduncum* at a concentration of 0.125 g/mL and indicates that there are highly significant differences (p=0.009). Figure 1B presents the results of pairwise comparisons of PLA2 activity of venom and total extract of *P. aduncum* and each of its fractions. The total extract of *P. aduncum* significantly (p<0.05) inhibited the PLA2 activity of the venom compared to its fractions.

**Table 4:** Inhibitory effect of *P. aduncum* extract and its fractions.

Treatment	Specific activity (SD)	p-value ^a^	Inhibition %
*L. muta muta* 0.125 mg/mL	3.69 (0.002)	0,009	NA
*P. aduncum* 0.125 g/mL	0.49 (0.004)		86.9
MF-1 0.125 g/mL	2.43(0.005)		34.3
MF-2 0.125 g/mL	1.21 (0.002)		67.1
MF-3 0.125 g/mL	1.67 (0.003)		54.9

MF-1: fraction 1 of *P. aduncum*, MF-2: fraction 2 of *P. aduncum*, MLF-3: fraction 3 of *P. aduncum*, SD: standard deviation, NA: not applicable.

a Kruskal Wallis test.

## DISCUSSION

Phenolic compounds able to inhibit PLA2 of the snake venom *L. muta muta* were detected in the extracts of *P. major* L., *P. aduncum* L. and their fractions, so they are related to inflammation. The total extract of *P. aduncum* and LLF-2 of *P. major* significantly inhibit PLA2 (p˂0.05) of the venom compared to the other samples evaluated.

The use of both plants for their anti-inflammatory effects is part of widespread ancestral knowledge. Our findings support this empirical knowledge, because PLA2 is the key enzyme in the inflammatory process. It is important to emphasize that this research is the first work carried out in the country on the subject; other works related to the inhibition of PLA2 of snake venoms by flavonoids isolated from plant species or commercial flavonoids aimed to study the mechanism of envenomation after an ophidian accident, which has not been the subject of this research ^5^^,^^10^^,^^11^.

In Peru, 1408 species used in traditional medicine have been described, and about 80% of the population is familiar with their empirical application in phytotherapy ^12^. These figures have led ethnomedicine to develop the exploration of bioactive components whose effectiveness has been reported by highland and jungle populations ^13^.

Although steroidal and non-steroidal anti-inflammatory drugs are currently used to treat acute inflammation, these drugs have not been successful in the treatment of chronic inflammatory disorders due to their side effects. Therefore, there is an urgent need to find safer anti-inflammatory compounds, such as those present in vegetables ^14^. Among the active components of extracts are flavonoids, a family of compounds whose members have many interesting biological properties, which could be studied to obtain new natural drugs ^15^.

Several studies have shown that plant extracts have anti-inflammatory activity both *in vitro* and *in vivo*^16^. Hussan *et al*. ^17^^)^ reported that aqueous, methanolic and ethanolic extracts of *P. major* leaves reduced inflammatory action both *in vitro* and *in vivo*. For the *in vitro* tests, they measured the concentration of the enzyme 11β-hydroxysteroid dehydrogenase type 1, and for *in vivo* tests, they used rats that were caused liver injury with acetaminophen. The results showed low concentrations of liver enzymes and proinflammatory cytokines, indicating a decrease in the inflammatory reaction.

A study on the lyophilized extracts of *P. major* prepared with water and with ethanol, used an *in vitro* model with H400 oral epithelial lines and reported that each extract, as well as their mixtures, had anti-inflammatory activity ^18^. Another research reported that 10 compounds were isolated from *P. aduncum* leaves, of which two showed inhibition of soluble epoxide hydrolase (SEH), an enzyme that regulates inflammatory processes in the brain during depression ^19^. In addition, Thao *et al*. ^20^^)^ caused inflammation in dendritic cells with proinflammatory cytokine lipopolysaccharides IL-12 p40, IL-6 and TNF-Alpha in order to study the inhibition of inflammation by *P. aduncum* fractions. They showed that seven of the isolated compounds inhibited IL-12 p40 and IL-6 production, but had no effect on TNF-alpha.

Research with extracts of five varieties of *Piper* on the immunomodulatory effect of inflammation in human monocytes showed that these samples amplified the anti-inflammatory response compared to ketoprofen, mainly reducing the production of IL-8 ^21^.

Although it is true that *P. major* and *P. aduncum* are used in Peru for their anti-inflammatory effects, research on the subject has been conducted in *in vivo* models with the total extract ^22^. In other countries, *in vitro* studies have been carried out with the compounds isolated from the total extracts of these species, using cellular and enzymatic models, being cyclooxygenases the most studied enzymes ^16^^-^^18^. The *in vitro* evaluation of PLA2 is of great importance, since this enzyme releases arachidonic acid from the cell membrane, thus initiating inflammation.

Interestingly, there are reports that show that extracts of *P. major* and *P. aduncum* have wound healing, antioxidant, analgesic, immunological and cytoprotective effects ^2^^,^^23^.

The mechanism of envenomation of the venom of *L. muta muta*, which contains PLA2, is highly complex and, therefore, has not been the subject of the present investigation. PLA2 activity has been used exclusively as a protein model because of its important participation in the initiation of the inflammatory process. Thus, by partially or totally inhibiting PLA2 with the extracts or their fractions, as shown in this study, the anti-inflammatory effect could be related to the presence of the phenolic compounds contained in these plants.

Flavonoids are phenolic compounds characterized by a chemical structure consisting of three rings called A, B, and C. Many reports indicate that these rings play an important role in the biological activities of these compounds, since they allow them to interact with specific proteins in intracellular signaling, generating inhibition or activation ^24^^,^^25^.

There are several articles on flavonoids with the ability to inhibit PLA2. Giresha *et al*. mentioned that quercetin is able to inhibit PLA2 from human and rabbit peritoneal neutrophils, as well as PLA2 from *Vipera russelli* snake venom and, to a lesser extent, PLA2 from porcine pancreas. Flavonols such as kaempferol, quercetin and myricetin were shown to be able to inhibit PLA2 from *V. russelli* venom, due to the unsaturation between the 2,3 carbons of its C-ring. However, the IC_50_ values of these flavonoids were at concentrations between 75 M-115 M, very high values even for pharmacological treatments ^25^.

Quercetin has been shown to modulate the PLA2 activity of the snake *Naja naja atra* due to interference between the enzyme and membrane phospholipids ^11^. In addition, it has been reported that PLA2 from the venom of the snake *Bothrops jararacussu* is inhibited by the flavonol rhamnetin, which causes an anti-inflammatory effect due to its A and C rings, which provide greater stability to the cell membrane ^5^. Toyama *et al*. ^10^, reported that quercetin decreased the catalytic activity of PLA2 from *Crotalus durissus terrificus* both *in vitro* and *in vivo*, by modifying the secondary structure of the enzyme and preventing coupling with the substrate.

On the other hand, Rodrigues *et al*. reported the presence of tannins in the leaves of *Laguncularia racemosa*, capable of inhibiting PLA2 isolated from the venom of *C. durisus terrificus*; these molecules had the ability to reduce edema and myonecrosis associated with inflammation. Our results only showed the presence of tannins in the LLF-2 fraction of *P. major* and this fraction had a great capacity to inhibit PLA2 of *L. muta muta* venom. The inhibition values we found were higher than those of the total extract, as well as the fractions that additionally contained flavonoids ^26^.

Some researchers have carried out fractionation of medicinal plant extracts in order to identify the chemical structure and evaluate the biological activity of the fractions. They emphasize that the compounds present in the total extracts have greater activity than the isolated compounds, so it is considered that there is synergism between these components.

Our results show that the total extract of *P. aduncum* has a greater capacity than its fractions to inhibit PLA2 of *L. muta muta* venom, in contrast to the total extract of *P. major*, where its fractions produce a greater degree of inhibition. In a previous study, total extracts of *O. rosea* “bloodsucker” were found to have a lower inhibitory capacity on thrombin than its fractions ^27^. In addition, Genc *et al.*^28^ evaluated the inhibition values of hyaluronidase, collagenase, and elastase enzymes by the aqueous extract of *P. major major* and three of its fractions (F-1, F-2 and F-3); it was evidenced that the total extract produced an inhibition of hyaluronidase and collagenase of 27.0% and 21.9%, respectively, while F-1 presented greater inhibition with values of 41.6% and 28.3% on both enzymes; elastase was not inhibited neither by the total extract nor by the fractions.

On the other hand, a study on the antioxidant effect of Ginko biloba extract and its four fractions, found that F-3 and F-1 together produced a greater antioxidant effect than each of them separately, which is evidence of synergism in this extract ^29^.

The study on mango (*Mangifera indica* L.) seeds showed that, of the twelve fractions, five had anticarcinogenic action to different degrees, but all had antioxidant action, the most potent and abundant being the butylated hydroxytoluene (BHT) fraction, used in the food industry for its high antioxidant power ^30^.

One of the limitations of our study was the lack of availability of adequate quantities of the fractions of the two studied species. This was due to the fact that only thin-layer chromatography was performed, which prevented carrying out tests to obtain the mean inhibitory concentration (IC_50_) both *in vitro* and *in vivo*. Nevertheless, we achieved the objectives of the research.

In conclusion, we found a significant inhibition of phospholipase A2 of *L. muta muta* venom by the total extracts of *P. major* and *P. aduncum*, being more potent that of *P. aduncum*. All the fractions obtained by TLC also showed ability to inhibit PLA2, but to different degrees. However, LLF-2, which contained only tannins, was the one that showed significant inhibition compared to its total extract. These PLA2 inhibitions by *P. major* and *P. aduncum* extracts are promising in the search for anti-inflammatory compounds.
