# Supplementary material for: In vitro anti-inflammatory activity of Plantago major L. and Piper aduncum L. on phospholipase A2 from the venom of snake Lachesis muta muta
Source: Rev Peru Med Exp Salud Publica. 2023 Sep 26;40(3):325–32. doi: 10.17843/rpmesp.2023.403.12191 (PMC10953663; doi:10.17843/rpmesp.2023.403.12191)
Supplement: Translation

# Translation: Actividad antiinflamatoria *in vitro* de *Plantago major* L. y *Piper aduncum* L. sobre la fosfolipasa A2 del veneno de la serpiente *Lachesis muta muta*

**Authors:** Mirtha Yarleque-Chocas, Flor Dorregaray-Llerena, Armando Yarleque-Chocas, Celso Gonzales-Chavesta

**Affiliations:** 1 Laboratorio de Investigación de Bioquímica y Principios Activos Naturales, Facultad de Medicina Hipólito Unanue, Universidad Nacional Federico Villarreal, Lima, Perú.; 2 Laboratorio de Recursos Fitogenéticos, Facultad de Ciencias Agrarias, Universidad Agraria del Ecuador, Milagro, Ecuador.; 3 Laboratorio de Biología Molecular, Facultad de Ciencias Biológicas, Universidad Nacional Mayor de San Marcos, Lima, Perú.; 4 Departamento de Estadística e Informática, Universidad Nacional Agraria la Molina, Lima, Perú.

**Keywords:** Extractos de Plantas, Plantago, Piper, Veneno, Fosfolipasa A2, Antiinflamatorio

## Abstract

**Objetivo.:**

Evaluar la actividad inhibitoria *in vitro* de los extractos de *Plantago major* «llantén» y *Piper aduncum «*matico» sobre Fosfolipasa A2 (PLA2) del veneno de la serpiente *Lachesis muta muta*.

**Materiales y métodos.:**

Esta investigación fue de tipo explicativa con diseño experimental. Se recolectaron hojas de *P. major* y *P. aduncum* en la provincia de Huarochirí en Lima, Perú. Se prepararon extractos alcohólicos diluidos en agua destilada y se realizaron los ensayos fitoquímicos, la cuantificación de fenoles y flavonoides, la cromatografía de capa fina (CCF) en celulosa y la actividad enzimática con PLA2. Se analizó la capacidad de inhibir la PLA2 con los extractos en estudio y sus fracciones. Para el análisis estadístico se utilizó la prueba de Kruskal Wallis y comparaciones múltiples de Bonferroni.

**Resultados.:**

Tanto en *P. major* como en *P*. *aduncum* se identificó cualitativamente la presencia de fenoles, flavonoides y taninos; además, *P. aduncum presentó* saponinas. La inhibición de la actividad de la PLA2 del veneno por el extracto total de *P. major* fue del 45,3%, y sus fracciones mostraron valores de inhibición: LLF-1 con 31,1%, LLF-2 con 66,3% y LLF-3 con 65,5%. En *P. aduncum,* los valores de inhibición para el extracto total fueron de 86,9%, y sus fracciones presentaron inhibiciones: MF-1 con 34,3%, MF-2 con 67,1% y MF-3 con 54,9%. El análisis estadístico demostró diferencias significativas en la inhibición de la PLA2 (p=0,009) por los extractos.

**Conclusión.:**

Los ensayos realizados demostraron una asociación entre el efecto antiinflamatorio de los extractos y la inhibición de la PLA2.

## INTRODUCCIÓN

El Perú es un país que cuenta con una gran riqueza en recursos fitogenéticos, teniendo solo en plantas vasculares un total de 19 147 especies. Esta diversidad permite que las plantas puedan relacionarse con los conocimientos tradicionales de las comunidades humanas del país, las que participan activamente en su conservación y uso sostenible ^1^. Las especies vegetales brindan diversos servicios ecosistémicos, y muchas de ellas aún no han sido estudiadas completamente, aunque su uso empírico es muy difundido, especialmente por sus propiedades medicinales.

En este contexto, *Piper aduncum* L., comúnmente conocida como «matico», es una especie nativa del Perú y está distribuida en varios departamentos, incluyendo Amazonas, Ayacucho, Cajamarca, Cuzco, Huánuco, Junín, Lambayeque, Lima, Loreto, entre otros ^2^. Por otro lado, *Plantago major* L., una especie introducida de Eurasia, se encuentra en zonas tropicales y subtropicales en todo el mundo, incluyendo Perú, donde se distribuye en todas las regiones geográficas y a altitudes entre 1500 y 2000 m ^3^.

Es interesante conocer los usos etnomedicinales de *P. aduncum* y *P. major* en el Perú, donde las hojas frescas o secas se utilizan en forma de infusión para tratar una variedad de problemas de salud, como resfriados, hongos, tos, heridas, bronquitis, fiebre, problemas renales, pulmonares, gástricos y respiratorios, así como para lavar heridas inflamadas ^4^.

En relación con las investigaciones sobre flavonoides y su actividad antiinflamatoria, se destaca la importancia de estudiar este efecto debido a que ciertos flavonoides, como la quercetina, contenidos en algunas plantas, pueden poseer propiedades antiinflamatorias. Bloquear la inflamación implica la capacidad de actuar sobre la actividad de enzimas involucradas en la síntesis de tromboxanos y leucotrienos, como la Fosfolipasa A2 (PLA2) y las ciclooxigenasas (COXI y COXII), así como tener un efecto inhibidor sobre la producción de óxido nítrico (NO), un mediador celular implicado en procesos fisiológicos y patológicos relacionados con la inflamación ^5^.

El veneno de la serpiente *Lachesis muta muta*, que habita en la selva peruana y es conocida como «shushupe», contiene una elevada actividad de PLA2. Por esta razón, la búsqueda de inhibidores sobre esta enzima se plantea como una alternativa para comprender el mecanismo del proceso antiinflamatorio ^6^. 

El objetivo de la presente investigación fue evaluar la actividad inhibitoria *in vitro* de los extractos de *P.major* «llantén» y *P. aduncum* «matico» sobre PLA2 del veneno de la serpiente *Lachesis muta muta*, ya que esta enzima juega un papel importante en el proceso inflamatorio*.* Es importante destacar que este estudio representa el primer aporte a nivel nacional en el conocimiento de la acción de los compuestos contenidos en estas plantas en la inhibición de PLA2, es decir, en su capacidad antiinflamatoria.

**MENSAJES CLAVE**

Motivación para realizar el estudio. *P. major* «llantén» y *P. aduncum* «matico» han sido utilizadas en la medicina tradicional por sus efectos antiinflamatorios y nuestra inquietud fue investigar esos efectos con relación a la enzima PLA2 que es la que inicia el proceso inflamatorio.

Principales hallazgos. Los extractos provenientes de las dos plantas inhiben a la PLA2 del veneno de serpiente, siendo el de *P. aduncum* más eficiente. Las fracciones mostraron inhibición en diferentes grados.

Implicancias. Los extractos de *P. major* y *P. aduncum* y sus fracciones muestran efecto inhibitorio in vitro sobre la PLA2 del veneno de serpiente, lo que se relaciona con el efecto antiinflamatorio.

## MATERIALES Y MÉTODOS

La investigación realizada fue de tipo explicativa y de diseño experimental. Se realizó en el Laboratorio de Investigación de Bioquímica y Principios Activos Naturales de la Facultad de Medicina «Hipólito Unanue» de la Universidad Nacional Federico Villarreal desde julio del 2021 a julio del 2022.

### Muestras vegetales

Las hojas de *Piper aduncum* L. y *Plantago major* L. utilizadas en la investigación fueron recolectadas entre agosto a octubre del 2019 en la provincia de Huarochirí en Lima, Perú. La localidad se encuentra a una altitud de 3044 m s. n. m. (11°50′41″S 76°23′02″O), con una temperatura que oscila entre 15 °C y 21 °C.

La identificación taxonómica de las especies se realizó en el Herbario de la Facultad de Ciencias Naturales de la Universidad Nacional Federico Villarreal, donde las muestras se encuentran depositadas con códigos de ingreso N.° 7400 para *Piper aduncum* L. y N.° 7399 para *Plantago major* L.

### Veneno de serpiente

El veneno liofilizado de la serpiente peruana *L. muta muta* usado en la investigación fue proporcionado por el Laboratorio de Biología Molecular, que tiene a cargo el «Serpentario Oswaldo Meneses» del Museo de Historia Natural de la Universidad Nacional Mayor de San Marcos en Lima, Perú. Los ejemplares de *L. muta muta* del serpentario proceden de la zona del Alto Marañón, en el departamento de Amazonas, y se mantienen en cautiverio, cumpliendo con las normas establecidas por el Servicio Nacional de Forestal y Fauna (SERFOR) del Ministerio de Agricultura y Riego.

### Extractos alcohólicos

Las muestras fueron hojas secas de cada especie. Se tomaron 200 g de cada muestra y se pulverizaron para aumentar su superficie de contacto con el solvente. Luego, estas muestras pulverizadas se colocaron en frascos de color ámbar para su maceración con etanol de 96° durante siete días. Después del periodo de maceración, se procedió a filtrar los extractos para eliminar las partículas sólidas y obtener un líquido clarificado. Posteriormente, se evaporó el solvente etanol en un equipo conocido como rotavapor hasta que los extractos quedaron completamente secos.

### Ensayos fitoquímicos cualitativos

Los ensayos fitoquímicos cualitativos realizados en la investigación para determinar la presencia de diferentes compuestos en los extractos de *Plantago major* y *Piper aduncum* fueron los siguientes:

Determinación de fenoles totales: se utilizó el reactivo de cloruro férrico para formar un complejo coloreado de color azul oscuro en presencia de compuestos fenólicos. Se agregó 1 mL de solución de la muestra en etanol al 70% y 2 gotas de cloruro férrico al 1% en un tubo de ensayo, y se observó el cambio de color ^7^.

Evaluación de flavonoides: se empleó la reacción de oxidación del magnesio metálico con el ácido clorhídrico concentrado para formar cloruro de magnesio que reacciona con los flavonoides y genera complejos coloreados. Se colocó 1 mL de solución etanólica de la muestra en un tubo de ensayo, se agregaron 10 gotas de HCl concentrado y virutas de magnesio metálico. Después de reposar por 5 minutos, se observó el color de la reacción ^7^.

Identificación de saponinas: las saponinas tienen la capacidad de romper la tensión superficial del agua y formar espuma. Se tomó 1 g de extracto seco al que se le añadió 10 mL de agua destilada, se calentó a 100 °C por 5 minutos y se agitó vigorosamente. La formación de espuma persistente a partir de 5 minutos fue calificada con cruces, indicando la presencia de saponinas ^7^.

Reconocimiento de taninos: los taninos tienen la capacidad de precipitar proteínas. Se colocó 1 mL de gelatina al 1% en solución salina en un tubo de ensayo y se agregaron 200 uL de la muestra disuelta en agua destilada. La presencia de precipitación de la proteína fue calificada como positiva, indicando la presencia de taninos ^7^.

### Cuantificación de fenoles

Para la reacción colorimétrica de óxido-reducción se utilizó el reactivo de Folin-Ciocalteu ^(^^8^. A 0,1 mL de cada muestra se agregó 1 mL de Folin-Ciocalteu al 10%, se dejó reposar por cinco minutos y se agregó 1 mL de carbonato de sodio al 7,5%. Posteriormente se colocaron los tubos por 15 minutos a baño maría (45°C), luego se leyeron a 765 nm en un espectrofotómetro Thermo Scientific Genesys 10. Los resultados fueron comparados con una curva de ácido gálico (Sigma Chemical), y expresados en mg/g de muestra. Se realizaron tres repeticiones con cada una de las muestras.

### Cuantificación de flavonoides

Los flavonoides totales fueron analizados por un método espectrofotométrico ^(^^9^. A 250 µL de cada muestra se le mezcló con 1000 µL de agua desionizadas, después se añadió 75 µL de NaNO_2_ al 20%, se dejó reaccionar cinco minutos para luego agregar 75 µL de AlCl_3_ al 10% y 500 µL de NaOH 1 M. La mezcla fue centrifugada a 3500 r.p.m. durante cinco minutos. Los flavonoides totales fueron expresados en mg catequina/g de muestra. Las absorbancias fueron medidas a 510 nm. Se realizaron tres repeticiones con cada una de las muestras vegetales.

### Actividad de PLA2

Se determinó midiéndose el tiempo de coagulación de una emulsión de yema de huevo al 45% con buffer Tris HCl 10 mM, cloruro de calcio 10 mM pH 7,4, luego de su incubación con 0,125 mg/mL del veneno de *L. muta muta* por 10 minutos a 37 °C ^6^.

Inhibición de PLA2 por los extractos

Preparación de las muestras: Se tomaron 1 mL del veneno de concentración 0,250 mg/mL y se mezcló con 1 mL de cada uno de los extractos vegetales por separado. Estas mezclas se preincubaron durante 10 minutos a una temperatura de 37 °C.

Preparación del sustrato: Se preparó una emulsión de huevo al 45% en buffer Tris HCl 10 M pH 7,4 con cloruro de calcio 10 mM, 1,5 mL de la emulsión se utilizó como sustrato.

Incubación con el sustrato: A 200 uL de la mezcla de veneno más extracto se le agregó al sustrato. Luego, esta mezcla se llevó a calentamiento a una temperatura de 100 °C.

Medición del tiempo de coagulación: Se midió el tiempo de coagulación de la yema de huevo en minutos después de la incubación con la mezcla de veneno más extracto.

Cálculo de la actividad específica: La actividad específica de la PLA2 se expresó como la variación del tiempo de coagulación de la yema por minuto dividido por el contenido de proteína del veneno en miligramos (U/mg).

### Cromatografía de capa fina en celulosa

Soluciones acuosas de 0,5 g/mL de los extractos *P. major* L. y *P. aduncum* L. fueron sembrados en las placas cromatográficas de celulosa, se empleó como fase móvil etanol: agua, 1:2 y 1:3, respectivamente.

### Análisis estadístico

Se utilizó el software R v.4.2.1 para analizar la existencia de diferencias significativas en la actividad inhibitoria sobre PLA2 entre los extractos totales y sus fracciones para las dos especies estudiadas, *P. major* (LLF-1, LLF-2 y LLF-3) y *P. aduncum* (MF-1, MF-2 y MF-3) respectivamente, mediante la prueba de Kruskal-Wallis, la cual es equivalente a un diseño completo al azar. De manera similar, para determinar cuál muestra presentaba una mayor actividad inhibitoria sobre PLA2, se llevaron a cabo comparaciones por pares usando los valores de actividad de PLA2 del veneno y de cada una de las muestras vegetales, y se aplicó la prueba de comparaciones múltiples de Bonferroni. Esta elección se basó en que no se cumplían los supuestos del análisis de varianza (normalidad en los errores y homogeneidad de varianzas) en ambos casos.

Para validar los resultados, se usó el software SPSS versión 28 y se generaron gráficos de comparaciones múltiples para brindar una explicación más detallada de las comparaciones entre las parejas de muestras.

### Aspectos éticos

El proyecto fue evaluado por el Comité de Ética de la Unidad de Investigación, Innovación y Emprendimiento de la Facultad de Ciencias Naturales y Matemática de la Universidad Nacional Federico Villarreal (código N.º 002-2022).

## RESULTADOS

### Detección de componentes bioactivos en los extractos totales

En la Tabla 1 se muestra el contenido de fenoles y flavonoides registrados en los extractos totales de *P. major* y *P. aduncum.* El contenido total de fenoles y flavonoides presentes en *P. major* fue menor en aproximadamente el 50% con respecto a *P. aduncum*.

**Tabla 1:** Concentración de fenoles totales y flavonoides en los extractos totales.

Extractos totales	Fenoles totales mg/g muestra (DE)	Flavonoides mg/g muestra (DE)
*P. major*	11,65 (0,037)	0,30 (0,018)
*P. aduncum*	21,12 (0,003)	0,73 (0,027)

DE: desviación estándar.

En cuanto al análisis cualitativo, se registró para los extractos de *P. major* y *P. aduncum* reacción positiva para fenoles, flavonoides y taninos; adicionalmente las saponinas se encontraron en el de *P. aduncum* (Tabla 2).

**Tabla 2:** Pruebas fitoquímicas de los extractos y las fracciones de *P. major* y *P. aduncum.*

Muestra	Fenoles	Flavonoides	Saponinas	Taninos	Rf
*P. major*	+	+	-	+	NA
LLF-1	+	+	-	+	0,58
LLF-2	+	-	-	+	0,70
LLF-3	+	+	-	+	0,88
*P. aduncum*	+	+	+	+	NA
MF-1	+	+	-	+	0,23
MF-2	+	+	-	-	0,58
MF-3	+	+	+	-	0,74

Presencia (+), ausencia (-), Rf: coeficiente de reparto, NA: no aplica.

LLF-1: fracción 1 de *P. major*, LLF-2: fracción de *P. major*, LLF-3: fracción 3 de *P. major.*

MF-1: fracción 1 de *P. aduncum*. LLF-2: fracción 2 de *P. aduncum*. LLF-3: fracción 3 de *P. aduncum*.

### Análisis de las fracciones de los extractos de *P. major* y *P. aduncum*

Las fracciones, procedentes de la CCF, de ambos extractos totales de P. *aduncum* y *P. major* fueron tres. Los valores de coeficientes de reparto (Rf) obtenidos para las fracciones de *P. aduncum* fueron de 0,23 a 0,74, mientras que, para las fracciones de *P. major* 0,58 a 0,88. En el análisis fitoquímico de las fracciones *de P. major* todas presentaron fenoles, flavonoides y taninos a excepción de LLF-2 que no presentó flavonoides. En las fracciones de *P. aduncum,* se registraron fenoles y flavonoides, adicionalmente en MF-1 se observó taninos y en MF-3 saponinas (Tabla 2). 

### Actividad inhibitoria de la PLA2 por los extractos de *P. major* y *P. aduncum*

Los resultados de los ensayos *in vitro* del efecto inhibitorio de la fosfolipasa A2 del veneno de *L. muta muta* por los extractos de *P. major* y *P. aduncum* mostraron variaciones significativas (p=0,009).

La Tabla 3 muestra el efecto inhibitorio de la PLA2 del veneno de *L. muta muta* por el extracto total de *P. major* a una concentración de 0,5 g/mL e indica que existen diferencias altamente significativas (p=0,009). Por otra parte, en la Figura 1A, en la comparación por pares de los valores de la actividad enzimática de PLA2 del veneno y los del extracto total de *P. major* y cada una de sus fracciones, se observó que LLF-2 inhibió significativamente (p<0,05) la actividad de la PLA2 del veneno en comparación con el extracto total y las otras fracciones.

**Figura 1:** Comparaciones multiples de la actividad enzimatica inhibitoria sobre PLA2 del veneno de *L. muta muta.*

**Tabla 3:** Efecto Inhibidor del extracto de *P. major* y sus fracciones.

Tratamientos	Actividad especifica (DE)	Valor de p ^a^	% Inhibición
*L. muta muta* 0,125 mg/mL	3,69 (0,002)	0,009	NA
*P. major* 0,5 g/mL	2,02 (0,002)		45,3
LLF-1 0,5 g/mL	2,55 (0,013)		31,1
LLF-2 0,5 g/mL	1,24 (0,002)		66,3
LLF-3 0,5 g/mL	1,28 (0,004)		65,5

LLF-1: fracción 1 de *P. major*, LLF-2: fracción 2 de *P. major*, LLF-3: fracción 3 de *P. major*, DE: desviación estándar, NA: no aplica.

a Prueba de *Kruskal Wallis.*

La Tabla 4 muestra el efecto inhibitorio de la PLA2 del veneno de *L. muta* muta por el extracto total de *P. aduncum* a una concentración de 0,125 g/mL e indica que existen diferencias altamente significativas (p=0,009). En la Figura 1B, se presentan los resultados de las comparaciones por pares de la actividad de PLA2 del veneno y del extracto total de *P. aduncum* y cada una de sus fracciones. El extracto total de *P. aduncum* inhibió significativamente (p<0,05) la actividad de la PLA2 del veneno en comparación con sus fracciones.

**Tabla 4:** Efecto Inhibidor del extracto de *P. aduncum* y sus fracciones.

Tratamientos	Actividad especifica (DE)	Valor de p ^a^	% Inhibición
*L. muta muta* 0,125 mg/mL	3,69 (0,002)	0,009	NA
*P. aduncum* 0,125 g/mL	0,49 (0,004)		86,9
MF-1 0,125 g/mL	2,43(0,005)		34,3
MF-2 0,125 g/mL	1,21 (0,002)		67,1
MF-3 0,125 g/mL	1,67 (0,003)		54,9

MF-1: fracción 1 de *P. aduncum,* MF-2: fracción 2 de *P. aduncum,* MLF-3: fracción 3 de *P. aduncum,* DE: desviación estándar, NA: no aplica.

a Prueba de Kruskal Wallis.

## DISCUSIÓN

En los extractos de *P. major* L., *P. aduncum* L. y sus fracciones se detectó la presencia de compuestos fenólicos capaces de inhibir la PLA2 del veneno de la serpiente L. muta muta, por lo que se los relaciona con la inflamación. El extracto total de *P. aduncum* y la LLF-2 de *P. major* inhiben significativamente a la PLA2 (p˂0,05) del veneno en comparación con las otras muestras evaluadas.

El conocimiento ancestral muy difundido es el uso de ambas especies vegetales por sus efectos antiinflamatorios, lo que estos hallazgos estarían respaldando los conocimientos empíricos, debido a que la PLA2 la enzima clave en el proceso inflamatorio. Es importante destacar que esta investigación es el primer trabajo que se realiza en el país sobre el tema; otros trabajos relacionados con la inhibición de PLA2 de venenos de serpientes por flavonoides aislados de especies vegetales o flavonoides comerciales están dirigidos al estudio del mecanismo de envenenamiento luego de un accidente ofídico, lo cual no ha sido motivo de esta investigación ^5^^,^^10^^,^^11^.

En el país se han descrito 1408 especies usadas en la medicina tradicional, y cerca del 80% de la población conoce su aplicación empírica la fitoterapia ^12^. Estas cifras han originado que la etnomedicina desarrolle la exploración de componentes bioactivos cuya efectividad fue observada por las poblaciones de sierra y selva ^13^.

Aunque los fármacos antiinflamatorios esteroideos y los no esteroideos se utilizan actualmente para tratar la inflamación aguda, estos fármacos no han tenido éxito en el tratamiento de trastornos inflamatorios crónicos debido a los efectos secundarios que presentan. Por lo tanto, existe una necesidad urgente de encontrar compuestos antiinflamatorios más seguros, como los que están presentes en los vegetales ^14^. Entre los componentes activos de los extractos están los flavonoides, una familia de compuestos cuyos miembros tienen muchas propiedades biológicas interesantes, que podrían ser estudiadas para obtener nuevos fármacos naturales ^15^.

Numerosas investigaciones han demostrado que extractos vegetales presentan actividad antiinflamatoria tanto *in vitro* como *in vivo* ^(^^16^. Hussan *et al*. ^17^ señalaron que los extractos acuosos, metanólicos y etanólicos de las hojas de *P. major* redujeron la acción inflamatoria tanto *in vitro* como *in vivo*. Para el primer caso, se midió la concentración de la enzima 11β-hidroxiesteroide deshidrogenasa tipo 1, y en el segundo caso*,* se utilizaron ratas a las que se les causó lesiones hepáticas con acetaminofén. Los resultados mostraron concentraciones bajas de las enzimas hepáticas y de las citocinas proinflamatorias, lo que indicando una disminución de la reacción inflamatoria.

Por otro lado, al estudiar los extractos liofilizados de *P. major* preparados uno con agua y el otro con etanol, en un modelo *in vitro* con líneas epiteliales orales H400, se observó que cada extracto, así como sus mezclas, poseían actividad antiinflamatoria ^18^. Asimismo, se ha publicado que se aislaron 10 compuestos de las hojas de *P. aduncum,* de los cuales dos presentaban inhibición del epóxido hidrolasa soluble (SEH), enzima que regula los procesos inflamatorios en el cerebro durante la depresión ^19^. Además, Thao *et al*. ^20^ estudiaron la inhibición de la inflamación por las fracciones de *P. aduncum,* utilizaron células dendríticas a las que causaron inflamación con los lipopolisacáridos de las citocinas proinflamatorias IL-12 p40, IL-6 y FNT-Alfa. El estudio demostró que, siete de los compuestos aislados inhibieron la producción IL-12 p40 e IL-6, pero no presentaron efecto sobre el FNT-Alfa.

Investigaciones con los extractos de cinco variedades de *Piper* sobre el efecto inmunomodulador de la inflamación en monocitos humanos demostraron que estas muestras amplificaron la respuesta antiinflamatoria en comparación con el ketoprofeno, reduciendo principalmente la producción de IL-8 ^21^.

Si bien es cierto que *P. major* y *P. aduncum* son usados en el Perú por sus efectos antiinflamatorios, las investigaciones sobre el tema se han realizado en modelos *in vivo* con el extracto total ^22^. En otros países, se han efectuado estudios *in vitro* con los compuestos aislados de los extractos totales de estas especies, utilizado modelos celulares y enzimáticos, siendo las ciclooxigenasas las enzimas más estudiadas^16^^-^^18^. La evaluación *in vitro* de la PLA2 es de gran importancia, ya que esta enzima que libera ácido araquidónico de la membrana celular, iniciando así la inflamación.

Es interesante señalar que existen informes que indican que los extractos de *P. major* y *P. aduncum* presentan efectos cicatrizantes, antioxidantes, analgésicos, inmunológicos y citoprotectores ^2^^,^^23^.

En cuanto al veneno de serpiente *L. muta muta*, que contiene PLA2, el mecanismo de envenenamiento es sumamente complejo y, por tanto, no ha sido motivo de la presente investigación. La actividad de PLA2 se ha empleado exclusivamente como una proteína modelo debido a su importante participación en el inicio del proceso inflamatorio. De esta manera, al inhibir parcial o totalmente la PLA2 con los extractos o sus fracciones, como se muestra en este estudio, se podría relacionar el efecto antiinflamatorio con la presencia de los compuestos fenólicos contenidos en estas plantas.

Los flavonoides son compuestos fenólicos que se caracterizan por poseer una estructura química constituida de tres anillos denominados A, B y C. Muchos informes señalan que estos anillos juegan un papel importante en las actividades biológicas de estos compuestos, ya que les permiten interactuar con proteínas específicas en la señalización intracelular, generando inhibición o activación ^24^^,^^25^.

Existen numerosas publicaciones sobre flavonoides con capacidad de inhibir a la PLA2. Giresha *et al*., mencionan que la quercetina es capaz de inhibir la PLA2 de los neutrófilos humanos y peritoneales de conejo, así como la PLA2 del veneno de la serpiente *Vipera russelli* y, en menor grado, la PLA2 del páncreas porcino. Los flavonoles como kaempferol, quercetina y miricetina mostraron ser capaces de inhibir a la PLA2 del veneno de *V. russelli*, debido a la insaturación entre los carbonos 2,3 de su anillo C. Sin embargo, los valores IC_50_ de estos flavonoides fueron de concentraciones entre 75 M-115 M, cantidades muy elevadas incluso para tratamientos farmacológicos ^25^.

La quercetina ha demostrado modula la actividad de la PLA2 de la serpiente *Naja naja atra* debido a la interferencia entre la enzima y los fosfolípidos de la membrana ^11^. Además, se ha observado que la PLA2 del veneno de la serpiente *Bothrops jararacussu* es inhibida por el flavonol ramnetina, lo que causa efecto antiinflamatorio gracias a la participación de sus anillos A y C, que le proporcionan mayor estabilidad a la membrana celular ^5^. En un estudio realizado por Toyama *et al*. ^10^, se demostró que la quercetina disminuyó la actividad catalítica de la PLA2 de *Crotalus durissus terrificus* tanto *in vitro* como *in vivo,* al modificar la estructura secundaria de la enzima e impedir el acoplamiento con el sustrato.

Por otro lado, Rodrigues *et al*., reportan la presencia de taninos en las hojas de *Laguncularia racemosa,* capaces de inhibir a la PLA2 aislada del veneno de la serpiente de *C. durisus terrificus;* estas moléculas tuvieron la capacidad de disminuir el edema y la mionecrosis asociados a la inflamación. En este contexto de la presente investigación, se detectó en la fracción LLF-2 de *P. major* únicamente la presencia de taninos y esta fracción mostró una gran capacidad para inhibir la PLA2 del veneno de *L. muta muta*. Los valores de inhibición encontrados fueron superiores a los del extracto total, así como a las fracciones que adicionalmente contenían flavonoides ^26^.

Algunos investigadores han realizado fraccionamiento de los extractos de las plantas medicinales con la finalidad de conocer la estructura química y evaluar la actividad biológica de las fracciones. Ellos destacan que los compuestos presentes en los extractos totales tienen mayor actividad que en los compuestos aislados, por lo que se considera que existe sinergismo entre estos componentes.

En el presente trabajo se ha mostrado que el extracto total de *P. aduncum* tiene mayor capacidad que sus fracciones para inhibir a la PLA2 del veneno de *L. muta muta*, a diferencia del extracto total de *P. major,* donde sus fracciones producen una inhibición en mayor grado. En un estudio previo, se observó que los extractos totales de *O. rosea* «chupasangre» tienen una menor capacidad inhibitoria sobre la trombina que sus fracciones ^27^. Además, Genc *et al*. ^28^ evaluaron los valores de inhibición de las enzimas hialuronidasa, colagenasa y elastasa por el extracto acuoso de *P. major major* y tres de sus fracciones (F-1, F-2 y F-3); se evidenció que el extracto total producía una inhibición de la hialuronidasa y la colagenasa de 27,0% y 21,9%, respectivamente, mientras que la F-1 presentó mayor inhibición con valores de 41,6% y 28,3% sobre ambas enzimas; la elastasa no fue inhibida ni por el extracto total ni por las fracciones.

Por otra parte, en una investigación en la que se midió el efecto antioxidante del extracto de *Ginko biloba* y sus cuatro fracciones, se encontró que las F-3 y F-1 juntas produjeron un mayor efecto antioxidante que cada una de ellas por separado, lo que es una evidencia de sinergismo en este extracto ^29^_._

El estudio sobre semillas de mango (*Mangifera indica* L.) demostró que, de las doce fracciones separadas, cinco tenían acción anticancerígena en diferentes grados, pero, todas presentaron acción antioxidante, siendo la más potente y abundante la fracción hidroxitolueno butilado (BHT), empleada en la industria alimentaria por su elevado poder antioxidante ^30^.

Esta investigación presentó limitaciones debido a la falta de disponibilidad de cantidades adecuadas de las fracciones de las dos especies estudiadas. Esto se debió a que solo se realizó cromatografía en capa fina, lo que impidió llevar a cabo pruebas para obtener la concentración inhibitoria media (CI_50_) tanto *in vitro* como *in vivo*. No obstante, se cumplió con los objetivos propuestos en el proyecto de investigación.

En conclusión, se observó una significativa inhibición de la Fosfolipasa A2 del veneno de *L. muta muta* por los extractos totales de *P. major* y *P. aduncum*, siendo más potente el de *P. aduncum.* Todas las fracciones obtenidas por CCF también mostraron capacidad de inhibir a la PLA2, pero en diferentes grados. No obstante, la LLF-2, que contenía únicamente taninos, fue la que presentó una inhibición significativa en comparación con su extracto total. Estas inhibiciones de la PLA2 por los extractos de *P. major* y *P. aduncum* son promisorias en la búsqueda de compuestos antiinflamatorios.
